# Copy number variants in locally raised Chinese chicken genomes determined using array comparative genomic hybridization

**DOI:** 10.1186/1471-2164-14-262

**Published:** 2013-04-17

**Authors:** Ming Tian, Yanqiang Wang, Xiaorong Gu, Chungang Feng, Suyun Fang, Xiaoxiang Hu, Ning Li

**Affiliations:** 1 State Key Laboratory for Agrobiotechnology, China Agricultural University, Beijing, 100193, China

**Keywords:** Copy number variation, Array comparative genomic hybridization, Duplication, Deletion, Locally raised Chinese chicken

## Abstract

**Background:**

Copy number variants contribute to genetic variation in birds. Analyses of copy number variants in chicken breeds had focused primarily on those from commercial varieties with nothing known about the occurrence and diversity of copy number variants in locally raised Chinese chicken breeds. To address this deficiency, we characterized copy number variants in 11 chicken breeds and compared the variation among these breeds.

**Results:**

We presented a detailed analysis of the copy number variants in locally raised Chinese chicken breeds identified using a customized comparative genomic hybridization array. We identified 833 copy number variants contained within 308 copy number variant regions. The median and mean sizes of the copy number variant regions were 14.6 kb and 35.1 kb, respectively. Of the copy number variant regions, 138 (45%) involved gain of DNA, 159 (52%) involved loss of DNA, and 11 (3%) involved both gain and loss of DNA. Principal component analysis and agglomerative hierarchical clustering revealed the close relatedness of the four locally raised chicken breeds, Shek-Ki, Langshan, Qingyuan partridge, and Wenchang. Biological process enrichment analysis of the copy number variant regions confirmed the greater variation among the four aforementioned varieties than among the seven other breeds studied.

**Conclusion:**

Our description of the distribution of the copy number variants and comparison of the differences among the copy number variant regions of the 11 chicken breeds supplemented the information available concerning the copy number variants of other Chinese chicken breeds. In addition to its relevance for functional analysis, our results provided the first insight into how chicken breeds can be clustered on the basis of their genomic copy number variation.

## Background

Genomic structural variation is an important and abundant source of genetic and phenotypic variation [1]. As a key type of genomic structural variation, copy number variant (CNV) is operationally defined as a DNA segment longer than 50 bp that is found in variable numbers relative to that found in a reference genome [2]. The different types of CNVs—duplications, deletions, insertions [2]—have different effects, which include changes in levels of gene expression, disruption of gene dosage, and loss of regulatory elements [3,4].

Array comparative genomic hybridization (aCGH) is an efficient and reliable method for analyzing changes in DNA copy number losses and gains. Since its invention in 1997 [5] and first used to examine DNA copy number in 2001 [6], aCGH technology has become an essential tool for identifying CNVs [7,8]. Since 2004, when two groups reported genome-wide analysis of human CNVs [9,10] and completion of a comprehensive human CNV map in 2006 [11], much of the attention focused on CNV research had been devoted to understanding human disease. Nonetheless, in addition to the large number of CNVs discovered in humans [12-15], considerable structural polymorphism had also been found for mouse [16], rat [17], macaque [18] and several domesticated animal genomes, including those of dogs [2,19], pigs [20], cattle [1,21-24], sheep [25], chickens [26-29], and horses [30].

Besides chickens, CNVs had been detected in other avian genomes, such as those of turkeys [26] and ducks [27]. Some commercial chicken breeds as Cobb Broiler, White Leghorn and also Chinese Dou had been reported for their CNV loci yet [29]. Herein, we reported the use of a genome-wide 400 K aCGH platform with custom-designed probes to map common CNVs in the genomes of 11 locally raised Chinese chicken breeds, besides the data of Cobb Broiler, White Leghorn and Chinese Dou, which had been reported previously under the same platform and reference sample [29]. We discussed the value of further cataloguing large amounts of such variations, some of which were likely to underlie breed-specific biology.

## Results and discussion

### Distribution of CNV loci and CNVRs in eleven chicken breeds

**Figure 1:**
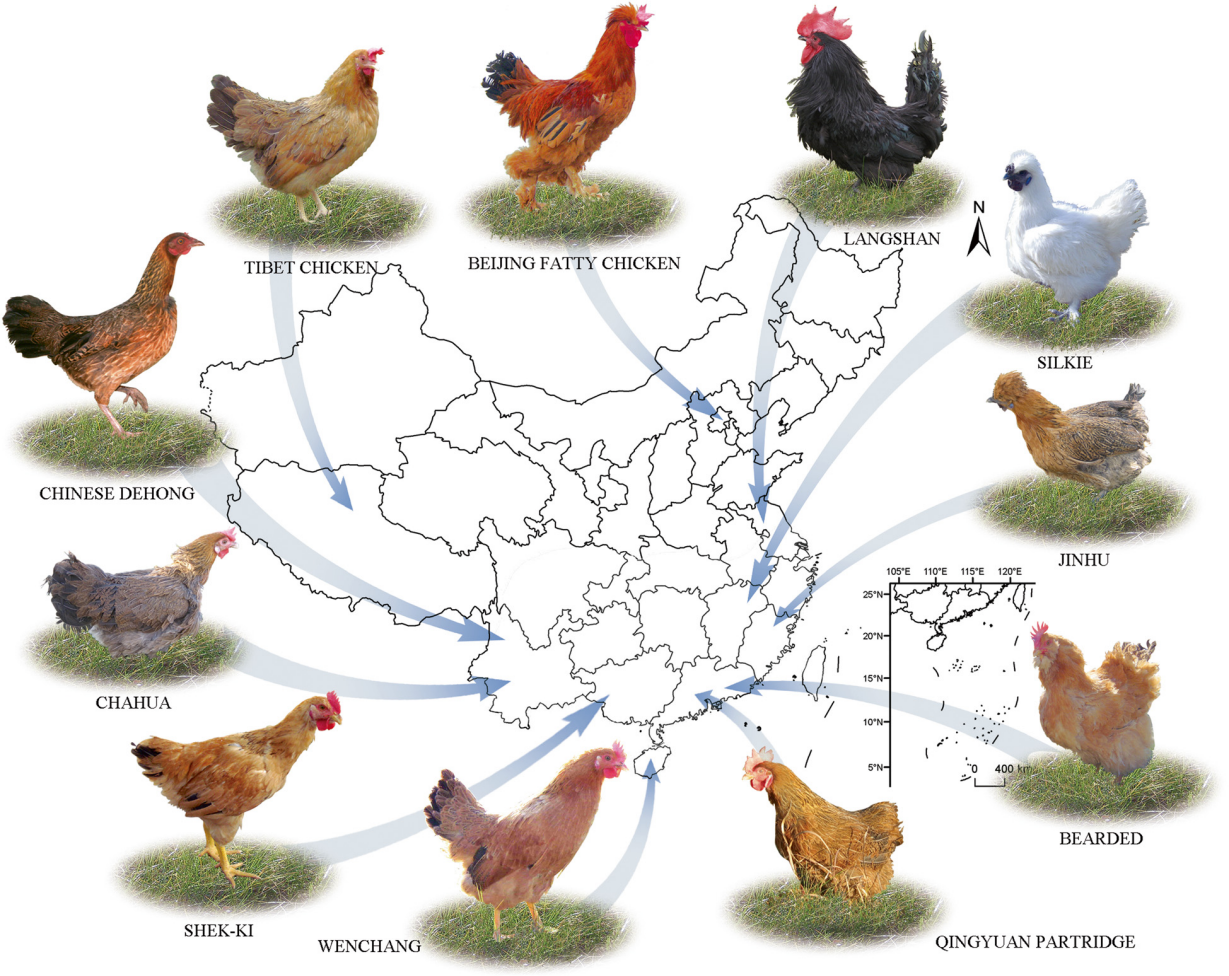
A global map showing the distribution of 11 various Chinese local chicken breeds.

The 11 chicken breeds (one male and one female in each breed) used in this study were the Silkie (WJ), Tibet (ZJ), Chahua(CH), Bearded (HX), Jinhu (JH), Anak (AK), Beijing fatty (BY), Langshan (LS), Qingyuan partridge (QY), Shek-Ki (SQ), and Wenchang (WC) varieties. Ten of these breeds originated in China, which has historically demonstrated extraordinary success in breeding disease-resistant chickens able to adapt to environmental changes (Table 1 & Figure 1). The AK variety was included as it represents several broiler chickens imported from Israel and was used in the breeding of the HuangYu variety, which is currently the fastest-growing domestic chicken breed in China. The genome of a female Chinese Dehong chicken, which was undomesticated in south of China was used as the reference for the aCGH experiment. The two animals in each breed were chosen randomly in their population. All the 11 chicken breeds belonged to preserved populations which were kept by our collaborator. And all the typical phenotypes for each breed were stable after breeding for several generations. Two individuals (one male and one female) were chosen in order to represent the typical breed and also to get rid of the gender-specific bias in our analysis as previous studies [31]. We used a high-throughput Agilent 2 × 400 K array CGH platform with custom-designed probes and excluded the sex chromosomes (chrZ and chrW) from our analysis to avoid gender-related analysis regarding global CNV regions. CNVs discovered from uncertain chromosomes (Chr#_random, ChrUn_random) and linkage groups that did not assigned to typical chromosomal loci (such as chrE22C19W28_E50C23 and chrE64) were also removed from the analysis. The rest array data used for further analysis were specific for chicken autosomes GGA 1–28 and GGA 32 to identify a total of 833 CNVs in the 11 chicken breeds (GGA 29, 30 and ~ 31 and GGA 33 ~ 38 were also excluded for their sequence data were not included in WUGSC2.1/galGal3 genome sequence). The mean and median lengths of the CNVs were 31.4 kb and 15.9 kb, respectively. The lengths of the CNVs ranged from 3.7 kb to 2 Mb. Within these segments, 402 CNVs involved an increase in DNA sequence, whereas 431 involved a decrease in DNA sequence. The total number of CNVs detected for each breed was 71 in WJ, 68 in ZJ, 57 in CH, 77 in HX, 64 in JH, 75 in AK, 68 in BY, 80 in LS, 83 in QY, 98 in SQ, and 92 in WC (Additional file 1–1). The average number of CNVs per breed was 36 in WJ, 34 in ZJ, 29 in CH, 39 in HX, 32 in JH, 38 in AK, 34 in BY, 40 in LS, 43 in QY, 49 in SQ, and 46 in WC. All of these CNVs localized to different sets of CNVRs in the genomes of the different chicken breeds.

**Table 1:** The origins and type of the 11 chicken breeds used in this study

**Name**	**Type**	**Origin**
Wenchang Chicken	layer/broiler	Hainan and Guangdong Province, China
Qingyuan partridge Chicken	broiler	Guangdong Province, China
Shek-Ki Chicken	broiler	Guangdong Province, China
Langshan Chicken	layer/broiler	Jiangsu Province, China
Anak Chicken	broiler	Israel
Jinhu Chicken	broiler	Fujian Province, China
Bearded Chicken	broiler	Guangdong Province, China
Beijing fatty Chicken	layer/broiler	Beijing, China
Silkie	broiler	Jiangxi and Fujian Province, China
Tibet Chicken	broiler	Tibet Province, China
Chahua Chicken	broiler	Yunnan Province, China
Chinese Dehong	wild	Yunnan Province, China

**Figure 2:**
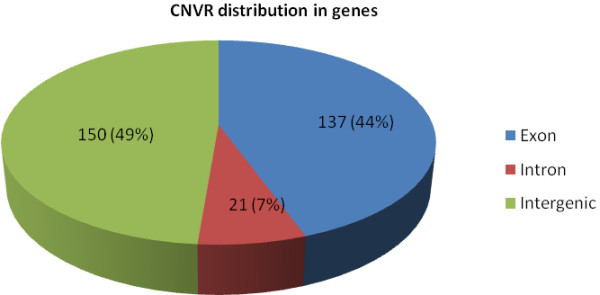
**Distribution of CNVRs in genes.** A total 308 of CNVRs were found in the autosomes, with 137 CNVRs (44%) in exons, 21 (7%) in introns, and 150 (49%) in intergenic regions.

A total of 308 CNVRs were characterized. The mean and median sizes of the CNVRs were 35.1 kb and 14.6 kb, respectively. Their lengths ranged from 5.8 kb to 2 Mb. The total number of CNVRs detected in each breed was 48 in WJ, 47 in ZJ, 42 in CH, 50 in HX, 49 in JH, 51 in AK, 47 in BY, 58 in LS, 66 in QY, 73 in SQ, and 72 in WC. Among these CNVRs, 198 (64%) were present in a single individual, 47 (15%) in two individuals, 19 (6%) in three individuals, 9 (3%) in four individuals, and 35 (12%) in more than four individuals. Whereas 138 (45%) CNVRs involved a gain of DNA, 159 (52%) involved a loss of DNA, and 11 (3%) involved both gain and loss of DNA (Additional files 2 and 3). In terms of their locations within genes, 44% of the CNVRs localized to exons, 49% localized to intergenic regions, and only 7% localized to introns (Figure 2). And in chicken whole genome data (WASHUC2.1/galGal3), nearly 3% of the genome localized to exons, 60% localized to intergenic regions, and 37% localized to introns (Additional file 4). It suggested that CNVRs were apt to happen in gene regions comparing with the whole genomic distribution.

### CNV loci and CNVRs among different chicken breeds

**Figure 3:**
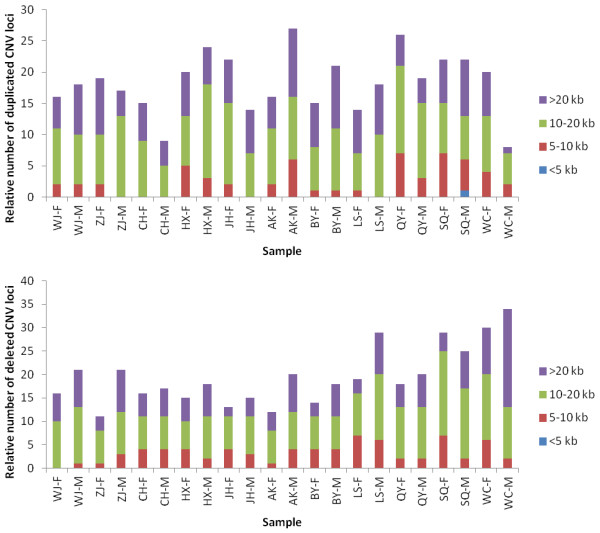
**Numbers of duplicated and deleted CNV loci in the 11 chicken breeds, classified according length.** The lengths of the CNV loci were classified as <5 kb, 5–10 kb, 10–20 kb, or >20 kb (indicated in blue, red, green, and purple, respectively). The upper panel displayed data for duplications, and the lower panel displayed data for deletions. The *x*-axis displayed the identities of the breed females (F) and males (M). For the duplicated CNV loci, the 10–20 kb- and >20 kb-length groups constituted 86% of the total. For the deleted CNV loci, the 10–20 kb- and >20 kb-length groups constituted 83% of the total.

**Figure 4:**
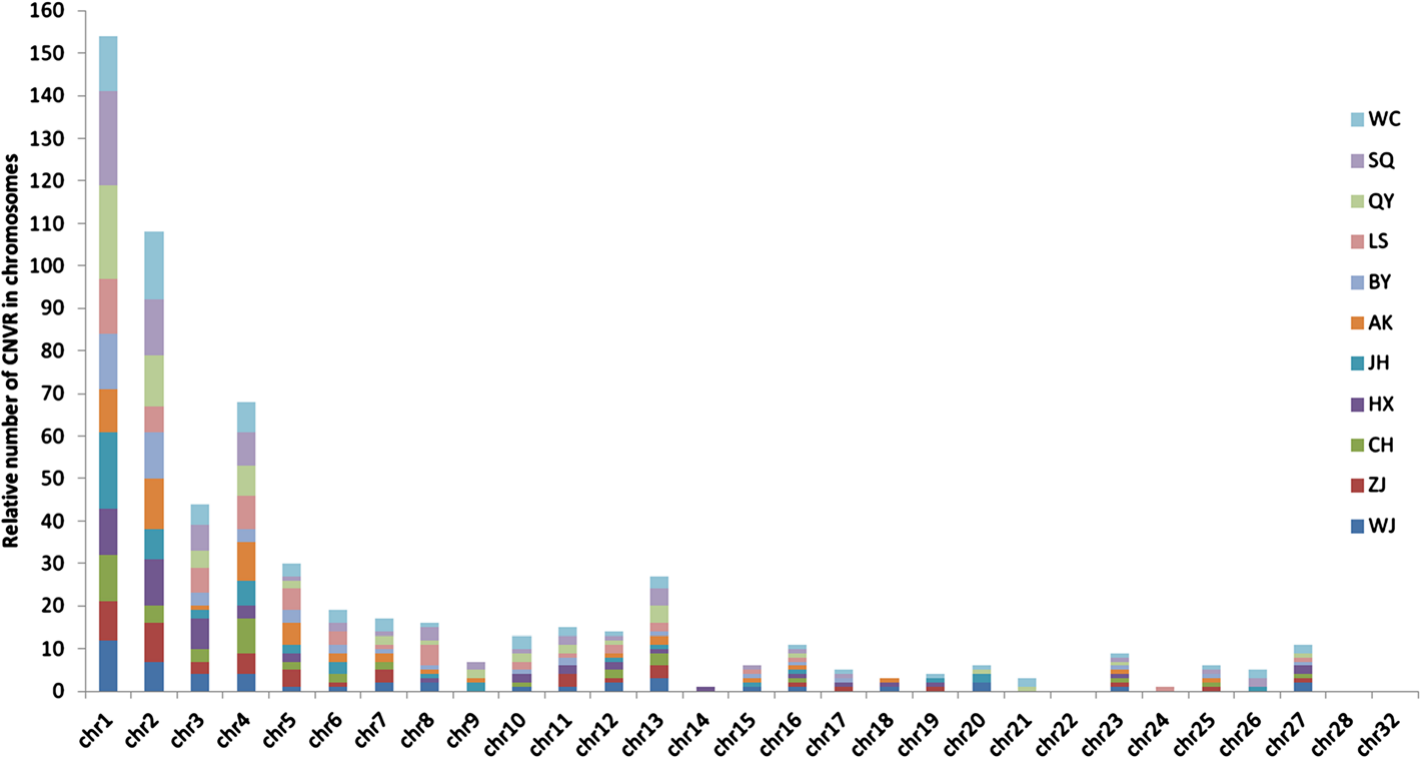
**Number of CNVRs in chicken chromosomes.** The chromosomal locations of the 308 CNVRs within the chicken autosomes (GGA1–28 and GGA32) for the 11 chicken breeds were identified by color. Each colored region represented both duplicated and deleted CNVRs for that breed on that particular chromosome.

Duplicated and deleted CNV loci for the chicken genomes were classified according to their lengths (Figure 3). For all breeds, the majority of the CNVs were larger than 10 kb. This category of CNVs included 86% of the duplicated loci and 83% of the deleted loci (Additional file 1–2 and 1–3). The relative numbers of the different CNVRs appeared to be distributed evenly within the autosomes of each breed, with the exception of chr22, chr28, and chr32, which did not contain any CNVRs (Figure 4). Of the 26 autosomes that contained CNVRs, in all breeds, chr1–chr5, chr13, and chr16 contained CNVRs, whereas the other chromosomes of each breed did not always have a CNVR.

Comparison of the CNVRs in the 11 breeds indicated that the average number of CNVRs shared among all the varieties was ~35 (standard deviation, 4), whereas the average number of variety-specific CNVRs was ~20 (standard deviation, 9). This means that the number of CNVRs found in each breed was approximately constant, whereas the variety-specific CNVRs differed in number (Table 2). In each breed, the number of variety -specific CNVRs was mainly contributed by individual variations. Compared with the other breeds studied, LS, QY, SQ, and WC had substantially more CNVRs and variety-specific CNVRs. This may indicated that these four breeds were more closely related to each other than to the other breeds.

**Table 2:** Inter-specific and intra-specific variation

**Breeds**	**Type**	**Number**	**Individual**	**Shared in breed**
WJ	variety-specific	16	13	3
	Shared	32	12	20
	Total	48	25	23
ZJ	variety-specific	10	10	0
	Shared	37	19	18
	Total	47	29	18
CH	variety-specific	11	9	2
	Shared	31	20	11
	Total	42	29	13
HX	variety-specific	13	10	3
	Shared	37	15	22
	Total	50	25	25
JH	variety-specific	20	20	0
	Shared	29	14	15
	Total	49	34	15
AK	variety-specific	15	14	1
	Shared	36	19	17
	Total	51	33	18
BY	variety-specific	14	11	3
	Shared	33	18	15
	Total	47	29	18
LS	variety-specific	25	22	3
	Shared	33	16	17
	Total	58	38	20
QY	variety-specific	30	28	2
	Shared	36	25	11
	Total	66	53	13
SQ	variety-specific	31	27	4
	Shared	42	23	19
	Total	73	50	23
WC	variety-specific	36	34	2
	Shared	36	22	14
	Total	72	56	16

### Principal component analysis

**Figure 5:**
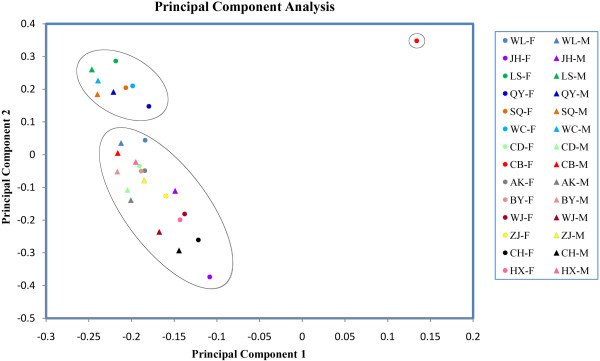
**Principal component analysis for 14 chicken breeds using whole-genome CGH log**_**2 **_**ratio data.**

To classify different clusters of breeds, we used whole-genome CGH log_2_ ratio data to perform principal component analysis (PCA, Figure 5). To complete this analysis, we included previously published data for Cobb Broiler (CB), White Leghorn (WL), and Chinese Dou (CD) chickens [29]. Using the PCA results, we could classify the 14 breeds (28 individuals) into roughly three categories. Whereas WC, LC, SQ, and QY clustered on the top left of Figure 5, the commercial A-hen division variety (CB–F) was found on the top right, and the remaining local breeds and commercial strains of the C-cock division (CB-M) clustered on the left of the bottom. In terms of regional distributions, the four varieties found on the top left of Figure 5—the WC, LS, SQ and QY varieties—all originated in the southern part of China. We proposed that the clustering of these four varieties reflected the admixture and inbreeding effects, and the influence of geographical proximity on the domestication process. As CB-F was a commercialized variety subjected to strong artificial selection pressure during its development, it was isolated from the other varieties in Figure 5.

### Agglomerative hierarchical clustering

**Figure 6:**
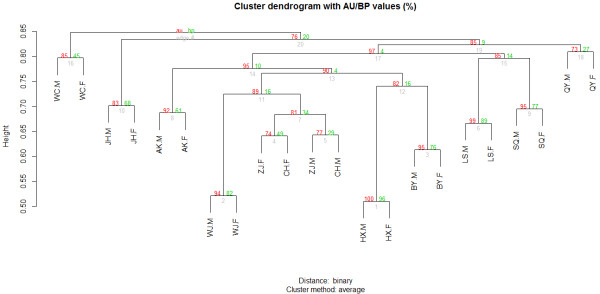
**Dendrogram generated by clustering individuals on the basis of their CNV similarities.** Numbers at the upper left of the node in red indicate the AU *p*-value, and numbers at the upper right of the node in green indicate BP values. Numbers beneath the nodes in gray are edge numbers.

To confirm the clustering patterns deduced by PCA and attempt an independent strategy for hierarchical clustering of chicken breeds on the basis of their ancestries, we performed a cluster analysis [32] for all individuals within each of the 11 breeds according to their absent or present CNVs. The cluster tree was shown in Figure 6. The approximate unbiased (AU) *p*-value and bootstrap probability (BP) value were shown for each node. Given that the AU *p*-value is less biased than the BP value, we focused on the AU *p*-values. Edge numbers, given beneath the nodes, represented the order in which the clusters were built. A smaller edge number indicated more closely related individuals. Although not all of the individuals from each variety group together, the overall trends were consistent with the clustering achieved using PCA, i.e.,WC, SQ, QY, and LS were more closely. The separate of the intra-specific individuals may caused by the limit that a CNV locus must contain five probes at least in the statistical analysis, which excluded some variety-specific CNVs.

### Functional analysis of CNVRs

We applied the BioMart webtool to the Ensembl *Gallus gallus* (WASHUC2) database to retrieve genes associated with the observed CNVRs. A total of 484 Ensembl genes were matched to 157 CNVRs (Additional file 2). Then, functional annotation and functional clustering analysis were performed for these genes (Additional file 5–1) using the CNVRs to identify biological processes enriched for CNVs [30,33,34]. Of 465 Ensembl gene identifiers found using the DAVID Functional Annotation Tool, 318 had functional annotations. Gene functional classification of the genes grouped 71 Ensembl genes into 8 clusters and excluded 249 unrelated genes from the analysis (Additional file 5–2). This indicated a wide distribution of CNVRs and reflected the inadequacy with which chicken genes had been annotated relative to other model mammalian species. The eight clusters corresponded to different categories in terms of the ontology of their molecular functions and biological processes, including structural components of the cytoskeleton, antigen processing and presentation, proteolysis involved in cellular protein catabolic processes, transition metal-ion binding, calcium-ion binding, G-protein-coupled receptor signaling, ATP binding, and DNA-dependent regulation of transcription. The gene clusters with the highest levels of significance were associated with structural components of the cytoskeleton (*p* = 5 × 10^–26^) and antigen processing and presentation (*p* = 9.9 × 10^–20^). To explore the basis of the distinct gene clustering in the groups comprising LS, QY, SQ, and WC, and the other Chinese local breeds, the DAVID Functional Annotation Tool was used again to analyze the CNVs of the two groups (Additional file 5–3 and 5–4). The results clearly indicated that the group comprising LS, QY, SQ, and WC had four more gene clusters than the group containing the other Chinese locally raised breeds, which means LS, QY, SQ, and WC were more closely related and uniform.

### Validation of CNVs by quantitative PCR

All real-time quantitative PCR (qPCR) assays were designed to confirm the presence of the CNVs detected by aCGH analysis. We chose to investigate 24 of the predicted CNVs in the QY and WJ breeds using one primer set per locus, included two CNVs adjacent to functional genes and also twenty-two random selected CNVs. Sixteen DNA samples from the two chicken breeds (eight from each breed), including the samples used in the aCGH analysis, were used for qPCR of the 24 CNVs (including 28 sets for selected loci and 2 sets for *PCCA* control. The four loci in chr1: 167926654–167954794, chr3: 107796818–107810140, chr13: 15750417–15766190 and chr23: 5943950–5959715 were detected in both QY samples and WJ samples, Additional file 6). Then we got the same results between aCGH and qPCR including 24 CNVs loci in sixteen individuals, which strongly supported our results that got from CGH arrays. *PCCA*, which encodes propionyl-coenzyme A carboxylase and was previously identified as a non-CNV locus, was used as the reference gene. Our findings confirmed the stability of *PCCA* in the chicken genome and its suitability as a reference gene to normalize sample amounts in different breeds. Most of the qPCR and aCGH results were concordant based on the detection in the same sample (except chr8: 27562722–27579422 and chr9: 19750362–19757329 loci, which were regarded as false positive and false negative loci). F-test were performed to check for homogeneity of variances between copy number of selected CNV loci and that of the reference *PPCA* locus at the first step. If the variances were statistically homogeneous, the Paired *T*-test was performed in the next step in order to check if the test samples showed significant mean difference comparing with the reference locus. As a result of that, 17 sets for F-test showed greater variance than references locus (p < 0.05). And for the rest 11 sets tested by Paired *T*-test, results indicated that they all showed the significant difference (p > 0.05) except one. Our results suggested that most of the test loci were truly CNVs. And for the chr2: 130453299–130471785 locus, it was detected and verified only in one sample of the Qingyuan partridge breed, which displayed rare CNV (details in Additional file 6).

Our aCGH analysis identified some interesting loci. One of the CNV loci locates on chr16 (positions 254,921–342,967 bp), which contains the gene that encodes the MHC class I antigen (YFV) [*ENSGALP00000040357*]. The gene, which is transcribed at a high level and is polymorphic, was duplicated in all of the breeds we studied, except for WC, in which it was deleted. Mature epitope-tagged YFV, which is associated with ®_2_-microglobulin, is located at the surfaces of chicken B (RP9) lymphoma cells [35]. CNVs occur predominantly in genes encoding components of the immune systems of birds [26], and mammals, such as humans [36] and Holstein cattle [37]. This trend was confirmed by our finding that the genes on chr16 of chicken, which encode components of the immune system, contained CNVs found universally in various breeds of chickens.

**Figure 7:**
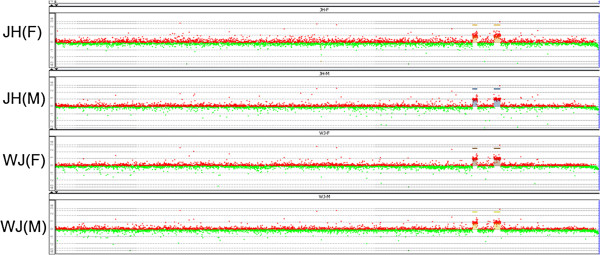
**CNV loci on Chr20 that was specific to the Jinhu and Silkie breeds.** The two specific duplicated CNV loci on chr20 in Jinhu (JH) and Silkie (WJ) male (M) and female (F) chickens occur at positions 10,718,139–10,844,289 bp and 11,263,937–11,435,137 bp.

The other specific duplication of a CNVR occurred on chr20 at positions 10,718,139–10,844,289 bp and 11,263,937–11,435,137 bp (Figure 7). The distance between the loci was 419.6 kb. The first region was associated with dermal hyperpigmentation in chickens [38] and contained four annotated functional genes, encoding endothelin 3 (*EDN3*), the ATP synthase epsilon subunit (*ATP5e*), the slowmo homolog 2 (*SLMO2*), and beta-1 tubulin (*TUBB1*). *EDN3* promoted melanoblast proliferation in chicken [39,40] as seen following ectopic expression of an *EDN3* transgene in mouse [41]. The three other aforementioned genes may also contributed to dermal hyperpigmentation or phenotypes associated with dermal hyperpigmentation. The second duplicated region did not contain any known coding or regulatory elements. We provided compelling evidence consistent with the conclusion reported by Dorshorst [42] and Shinomiya [43] that a complex genomic rearrangement (duplication) on chr20 involving the *EDN3* locus caused the hyperpigmentation associated with fibromelanosis in the chicken. The genetic causative mutation of fibromelanosis was an inverted duplicated and junction of two genomic regions separated by 417 kb in wild-types. One duplicated regions contained *EDN3*, the expression level of which gene was increased during embryo developmental stages in Silkie and also maintained a highly expressed level especially in adult skin [42]. Furthermore, given that WJ and JH were the only two breeds with this CNVR, we concluded that the dermal hyperpigmentation of these two Chinese local chicken breeds was also caused by the copy number variation of this CNVR. On the other hand, WJ and JH were closely distributed in the southeast of China. So it could be supposed that these two breeds originated from the same place or it might also be due to the trait being purposely bred into different strains.

We also found one other breed specific CNVR in AK, two in CH, three in WJ, three in SQ, three in LS, two in QY, two in WC, three in BY, and three in HX. To associate CNVRs with specific phenotypes, the genes in the intra-specific CNVRs of BY and HX were selected for further analysis. There were two genes in the three CNVRs of BY. One of these genes encoded the mitochondrial ribosomal protein L15 (*MRPL15*) [44], and the other encoded the homologous *Homo sapiens* solute carrier family 25 (mitochondrial carrier, brain) member 14 protein (*SLC25A14*) [45]. Neither of them appeared to be closely associated with macroscopic features of BY, such as feathered feet.

In HX, five genes appeared in the three HX breed specific CNVRs. The CNVR on chr2 between positions 147,922,354 bp and 147,936,835 bp contained no genes, and between them, two CNVRs on chr27 (positions 1,607,367–1,629,996 bp and 4,125,095–4,156,647 bp) had five genes. Among them, the gene that encoded keratin 222 (*KRT222*; positions 4,153,995–4,159,597 bp) attracted our attention, owing to the participation of the keratin family in development of the hair follicle and epithelium [46]. It seemed possible that duplication of *KRT222* may (at least partially) account for the bearded phenotype of HX chickens, although further work is necessary to evaluate this speculation.

## Conclusion

This was the first study to investigate the CNVs in locally raised Chinese chicken varieties by using customized CGH array. It described the distribution of CNVs and compared the differences in the CNVRs of the 11 chicken breeds. Our results supplemented the information available for chicken CNVs and provided the first insights into how chicken breeds cluster on the basis of CNVs, which will be valuable for elucidating the relationship between CNVRs and regulatory mechanism of chicken functional genes.

## Methods

### Ethics statement

All animal work was conducted according to the guidelines for the care and use of experimental animals established by the Ministry of Science and Technology of the People’s Republic of China (Approval number: 2006–398). The blood samples of chickens were collected from the brachial vein by standard venipuncture procedure approved by the Animal Welfare Committee of China Agricultural University (Permit Number: XK622).

### Chicken breeds and DNA isolation

All native chicken breeds were obtained from the Poultry Institute of Jiangsu province, Chinese Academy of Agricultural Sciences, and the Chinese Dehong variety, which was a strain of red jungle fowl from South China, was obtained from the Wild Animal Rescue Shelter Center of Yunnan province (Table 1 & Figure 1). Thirty-nine chickens from the 12 breeds (two of each breed, except for eight each of the Qingyuan partridge and Silkie varieties) were used. The Qingyuan partridge and Silkie breeds were each represented by four males and four females, whereas the other 10 breeds were each represented by one male and one female. The DNA of a female Chinese Dehong chicken was used as the reference. Blood samples were collected and stored at -20°C until DNA was extracted using Wizard Genomic DNA Purification kit reagents (No. A1125; Promega, USA). All DNA samples were analyzed using agarose gel electrophoresis and spectrophotometry, and DNA concentrations were measured using a NanoDrop 2000 instrument (Thermo Fisher Scientific, Waltham, MA, USA). DNA from 23 chicken samples (one male and one female from each of the 11 breeds plus the DNA from the reference chicken) was analyzed using aCGH, and DNA from the eight Silkie and eight Qingyuan partridge chickens were analyzed using qPCR.

### High-density array CGH design

The microarrays used for comparative genome analysis were designed and produced by Agilent Technologies and synthesized *in situ* as 60-mer oligonucleotide arrays as described [29]. We used an Agilent 2 × 400 K custom-designed high-density microarray and 420,288 probes. The Agilent High-Density Probe database was used to select 414,111 experimental probes from more than 4 million validated chicken CGH probes, plus 4,545 Agilent positive control probes, 1,182 Agilent negative control probes, and 450 probes located in areas that were not included in the database. The microarray covered the 29 autosomes, the 2 sex chromosomes, and 25 randomly selected chromosomal fragments. The probes covered exonic, intronic, and intergenic regions of the genome, which were each uniquely represented in the 2006 (WUGSC2.1/galGal3) version of the chicken genome.

### Hybridization and scanning

All array hybridizations were performed according to the manufacturer’s recommended protocols. For hybridization, DNA was first digested with *Alu*I and *Rsa*I, and then fluorescently labeled using Agilent Genomic DNA Labeling kit PLUS reagents (No. 5188–5309; Agilent Technologies, Santa Clara, CA, USA). After using standard procedures to label genomic DNA that included cyanine 5'-dUTP for test samples and cyanine 3'-dUTP for reference samples, we used an Amicon Ultra-0.5, Ultracel-30 Membrane system (30 kDa; No. UFC5030BK; Millipore, Billerica, MA, USA) to purify the labeled DNA fragments. Specific activity was calculated by measuring the absorbance at 260 nm (DNA), 550 nm (cyanine 3), or 650 nm (cyanine 5). Array hybridization was performed using Agilent Oligo aCGH Hybridization kit reagents (No. 5188–5380) for 40 h at 20 rpm in a 65°C Agilent hybridization oven. Arrays were scanned at 3-μm resolution using an Agilent scanner, and Agilent Feature Extraction software was used for image analysis (version 10.7, with 90% laser power value and 100% PMT).

### Statistical analysis

The statistical analysis for CNV interval detection was performed using Agilent Genomic Workbench Standard Edition 6.5 software. The Aberration Detection Method 2 algorithm was used to identify genomic variation given the log_2_ ratio of fluorescent signals between test and reference DNA samples [47]. The QC metrics motif of the Agilent workbench software ensured adequate quality control of the hybridization data. To be included in the analysis, an array signal needed to have an intensity value >50 and a signal-to-noise ratio >30. A relatively stringent calculated threshold of six was used in the analysis to minimize the numbers of false positives. Aberrant segments were called for a CNV locus when the average log_2_ ratio was greater than | ± 0.5| and also contained at least five probes. Fuzzy zero correction prevented inclusion of aberrant segments with low average log_2_ ratios. The raw data of our custom-designed aCGH experiments and the sequence information of our probes have been deposited into the GenBank GEO database (GSE36504). http://www.ncbi.nlm.nih.gov/geo/info/linking.html.

Statistical analysis for qPCR results were performed using SPSS software (version 17.0) for F-test and Paired *T*-test analysis.

### Confirmation by qPCR

Primers for real-time qPCR, designed using Primer Express 2.0 software (Applied Biosystems, Carlsbad, CA, USA), were used to amplify fragments each ~100 bp in length that were positioned within each selected CNV locus. Standard curves were plotted using measurements taken for different concentrations of standard DNAs. The primers for *PCCA* were as described [29]. The BLAT web tool, accessed at the University of California, Santa Cruz website (http://genome.ucsc.edu/cgi-bin/hgBlat?command=start), showed that the sequences were specific for each region of interest. Melting curve and amplification analyses validated the primers. The qPCR reactions were carried out as follow: The thermal cycles comprised 1 cycle of pre-incubation at 95°C for 5 min, 40 cycles of amplification (95°C for 10 s, 60°C for 10 s, and 72°C for 10 s), and a final dissociation step (95°C for 5 s, 60°C for 1 min, and 97°C for 5 s). Each genomic DNA sample was diluted into double distilled water to 10 ng/μl, with the concentrations verified using a NanoDrop instrument. A standard curve was prepared by taking the average of triplicate measurements for reference Chinese Dehong genomic DNA at five concentrations (40, 20, 10, 5, and 2.5 ng/μl) in the same plate as the test samples. SYBR Green–based real time qPCR assays were performed using a Roche LightCycler480 instrument with a 96-well block (Roche Applied Science, Indianapolis City, IN, USA). All qPCR samples were assayed in quadruplicate. Each reaction contained 10 ng of template, and all results were analyzed using LightCycler480 software 1.5 (Roche Applied Science, Indianapolis City, IN, USA) with a Ct threshold of 0.2. Relative copy numbers were assigned by comparing the Ct values with the standard curve and the number of copies in 1 ng of reference DNA (arbitrarily defined as one unit).

### PCA

PCA analysis was performed using the PCA analysis function in the Golden HelixTree software package (Golden Helix, Inc. Bozeman, MT, USA). The log_2_ ratio data of all probes were used in PCA except the sexual chromosome and controls. Cobb Broiler, White Leghorn and Chinese Dou were added which got from the same custom-designed Agilent 2 × 400 K array with the same reference sample [29].

### Clustering analysis

To group the 22 individuals according to their CNV similarities, we built a scoring matrix of the CNVR data for each individual. As the majority of CNVRs were observed only gains or only losses at the same locus among these breeds (rather than included both gains and losses), binary measure distances would be a good reflection of our data, which endowed the same weight to “zero” elements and “non-zero” elements. So if the CNVR of the loci showed absence, we encoded a value of “0” with the locus; otherwise, if the CNVR of the loci was presence (either gain or loss), we encoded “1” with it [32]. A hierarchical agglomerative clustering was applied to the matrix composed of the individual vectors using the pvclust function in the pvclust R package. Pvclust was an add-on package to the R Statistical Software for the assessment of the uncertainty in a hierarchical cluster. Multiscale bootstrap resampling was used to calculate the AU *p*-value, which was less biased than the BP value calculated by the conventional bootstrap resampling method [48]. We used an unweighted pair-group average calculation for agglomeration. The robustness of each branch was 10,000 bootstraps, and a hierarchy for the individual elements was built successively, according to the chosen distance and starting with the two closest elements in each case.

### Functional annotation of the clustering analysis

We used the Ensembl *Gallus gallus* (WASHUC2) BioMart webtool to retrieve genes associated with the observed CNV loci. These Ensembl genes identifiers could be imported and accepted by the DAVID Bioinformatics Resources 6.7 (http://david.abcc.ncifcrf.gov/). We used the basic “functional annotation clustering” and “gene functional classification” tool to cluster gene ontology terms of the input genes into functional related groups. When performing the classification analysis, we applied the medium classification stringency, implemented Benjamini multiple testing correction and set the threshold value of Enrichment score at 1.0 [34]. The data of gene functional classifications were processed by Microsoft Excel in order to determine the enrichment for biological processes.

## Abbreviations

CNV: Copy number variants; aCGH: Array comparative genomic hybridization; CNVR: Copy number variants region; PCA: Principal component analysis; AU: Approximate unbiased; BP: bootstrap probability; qPCR: quantitative polymerase chain reaction; EDN3: Endothelin 3; QC: Quality control; GEO: Gene expression omnibus

## Competing interests

The authors have declared that no competing interests exist.

## Authors’ contributions

XH and NL designed and guided the project; MT conducted the experiments and analyzed the data; YW assisted with the bioinformatics and data analysis; XG, CF and SF helped analyze the data; and MT, YW, XH and NL wrote the manuscript. All authors have read and approved the final manuscript.

## Supplementary Material

Additional file 1 CNVs detected in each sample and the number of duplicated and deleted CNV loci. Click here for file

Additional file 2 CNVRs overlap in gene content. Click here for file

Additional file 3 The distribution of CNVRs in individuals, including the proportion of CNVRs involving a gain of DNA and the proportion involving a loss of DNA. Click here for file

Additional file 4 The distribution of exon, intron and intergenic regions in chicken genome (GGA1-28 and GGA32). Click here for file

Additional file 5 **Biological processes enriched in CNVRs.** Additional file 5–1 Clustering of CNVRs with an Ensembl ID as identified using the DAVID Functional Annotation tool. Additional file 5–2 Classification of gene functions for all Ensembl ID genes from Additional file 5–1 annotated by DAVID. Additional file 5–3 Classification of gene functions for the LS, QY, SQ, and WC breeds. Additional file 5–4 Classification of gene functions for the locally raised Chinese chicken breeds other than LS, QY, SQ, and WC. Click here for file

Additional file 6 **Validation by qPCR for the 24 loci in QY and WJ and the summary of the statistical analysis qPCR results.** A total of 16 samples for each of the two breeds were analyzed using qPCR for 24 loci. Each sample DNA was adjusted to 10 ng/μl using a NanoDrop 2000 instrument. The QY-F, -5, -7, -9 and WJ-F, -30, -33, -35 samples were from females, whereas the QY-M, -52, -91, -93 and WJ-M, -61, -62, -71 samples were from males. The QY-F, QY-M, WJ-F, WJ-M samples were the same as those used in the aCGH analysis. Click here for file
