# Supplementary figures and images for: Copy number variants in locally raised Chinese chicken genomes determined using array comparative genomic hybridization
Source: BMC Genomics. 2013 Apr 17;14:262. doi: 10.1186/1471-2164-14-262 (PMC3637819; doi:10.1186/1471-2164-14-262)

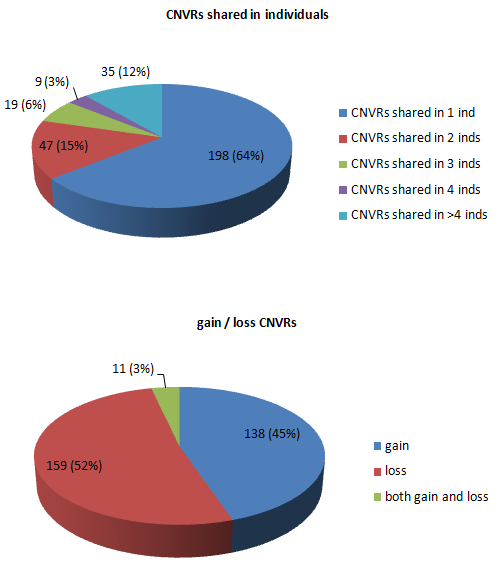

Supplement: Additional file 3 — The distribution of CNVRs in individuals, including the proportion of CNVRs involving a gain of DNA and the proportion involving a loss of DNA. [file 1471-2164-14-262-S3.tiff]
