# Supplementary material for: Copy number variants in locally raised Chinese chicken genomes determined using array comparative genomic hybridization
Source: BMC Genomics. 2013 Apr 17;14:262. doi: 10.1186/1471-2164-14-262 (PMC3637819; doi:10.1186/1471-2164-14-262)
Supplement: Additional file 6 — Validation by qPCR for the 24 loci in QY and WJ and the summary of the statistical analysis qPCR results. A total of 16 samples for each of the two breeds were analyzed using qPCR for 24 loci. Each sample DNA was adjusted to 10 ng/μl using a NanoDrop 2000 instrument. The QY-F, -5, -7, -9 and WJ-F, -30, -33, -35 samples were from females, whereas the QY-M, -52, -91, -93 and WJ-M, -61, -62, -71 samples were from males. The QY-F, QY-M, WJ-F, WJ-M samples were the same as those used in the aCGH analysis. [file 1471-2164-14-262-S6.doc]

**Validation by qPCR for the 24 loci (including 28 sets and 2 sets for *PCCA* control) in QY and WJ and the summary of the statistical analysis qPCR results.**


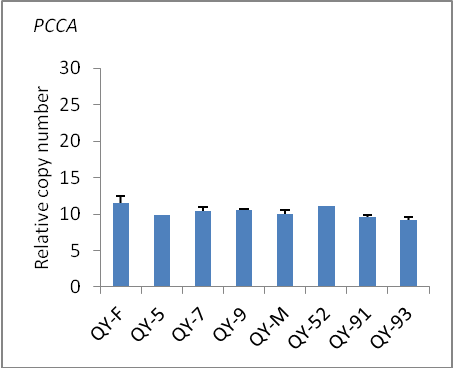

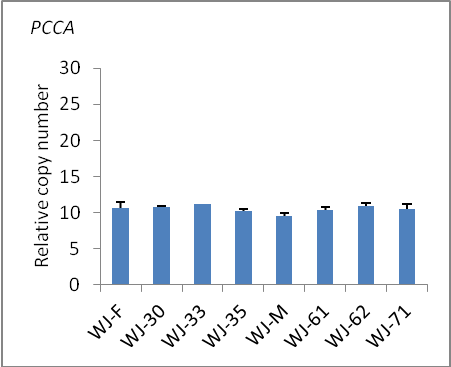


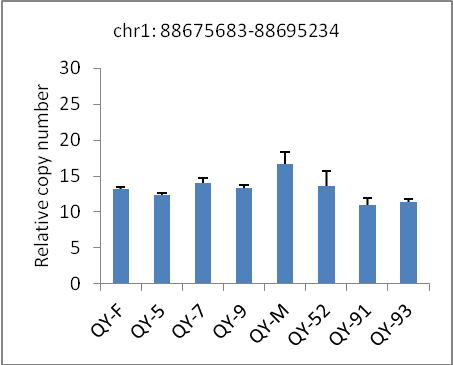

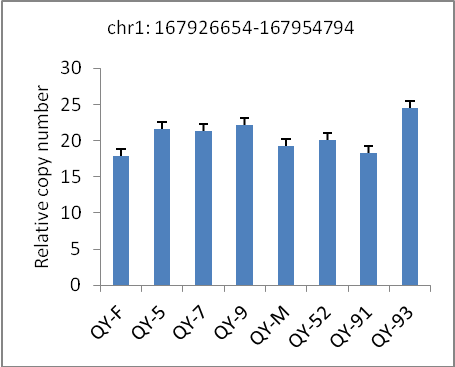


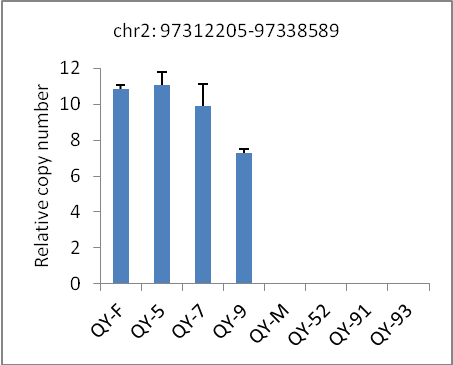

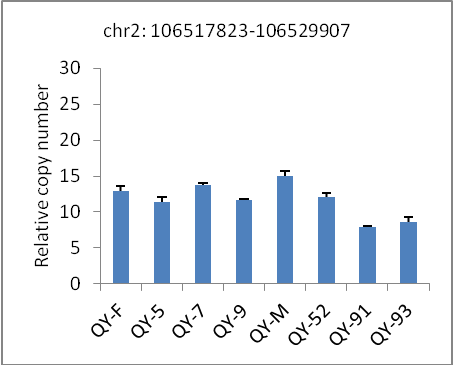


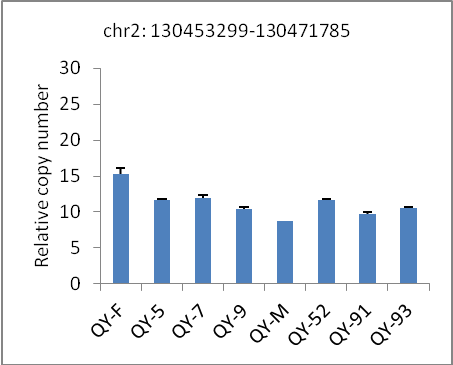

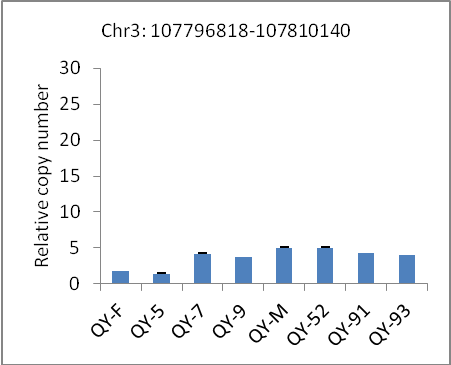


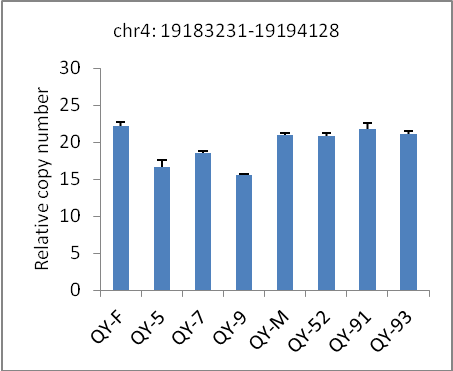

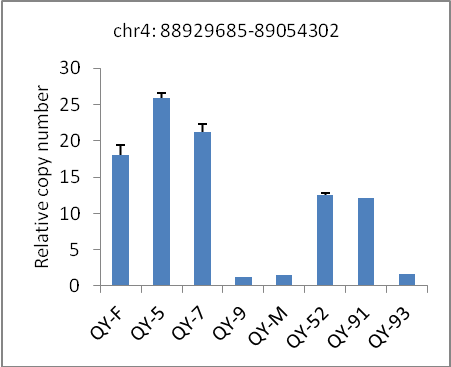


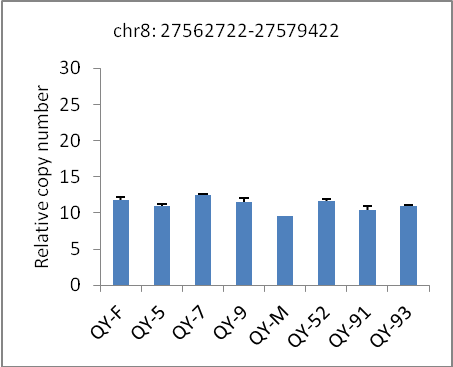

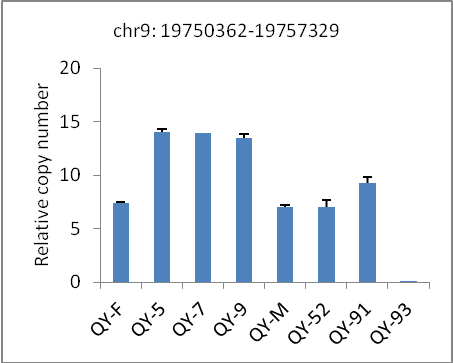


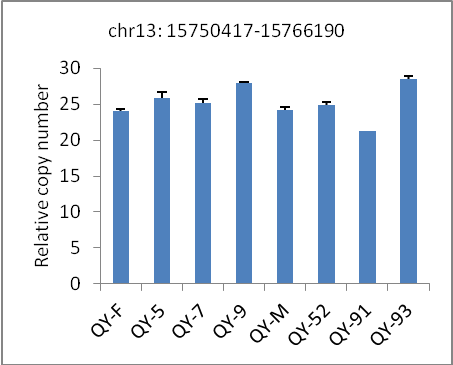

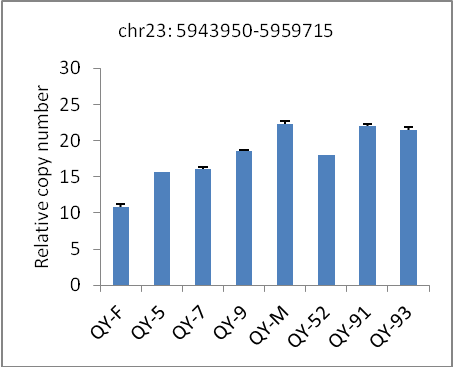


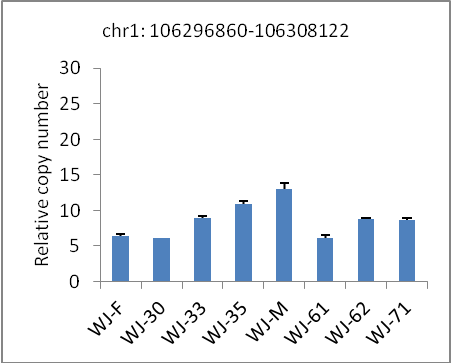

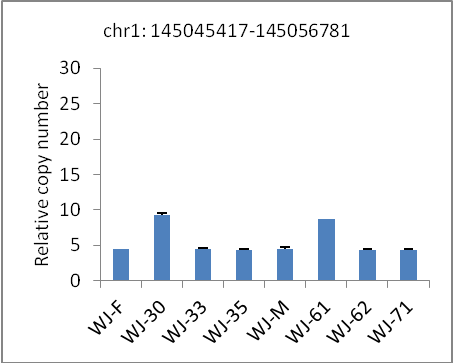


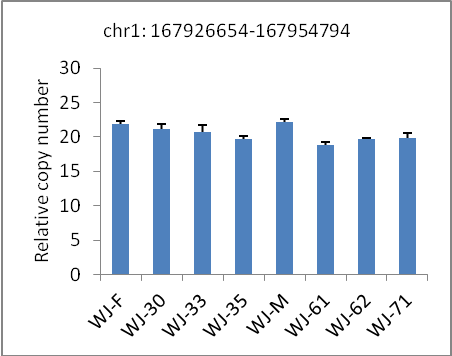

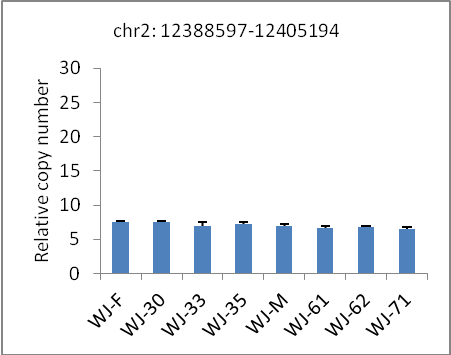


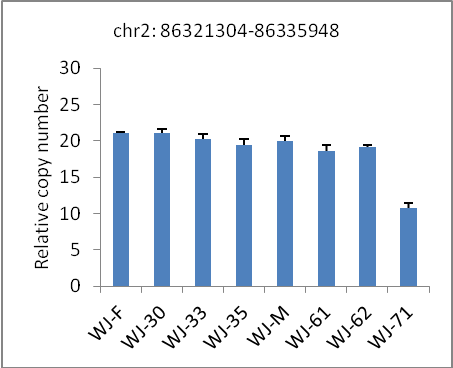

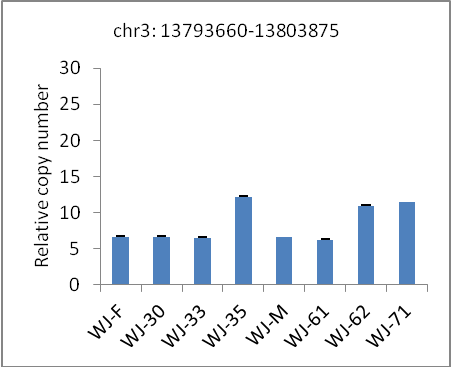


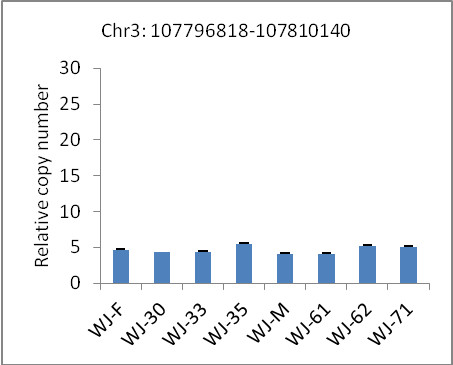

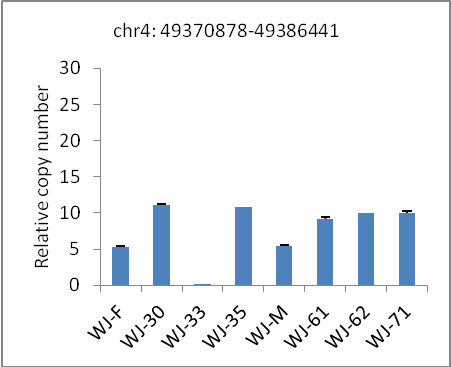


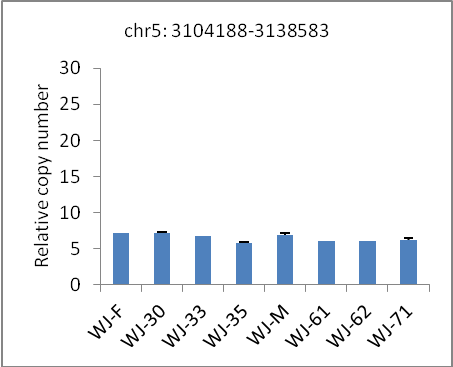

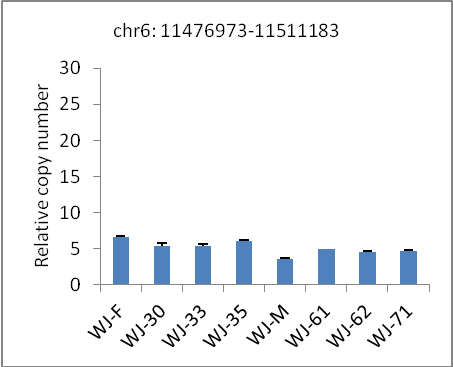


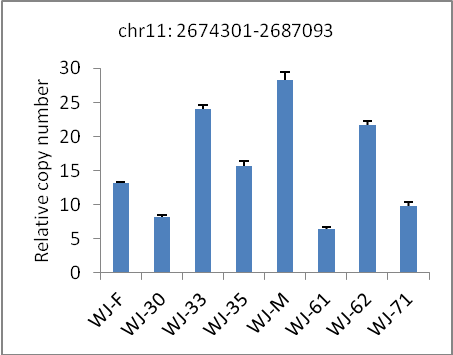

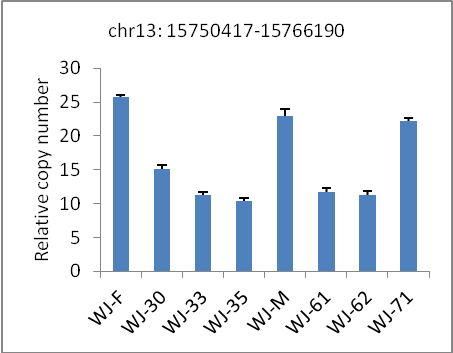


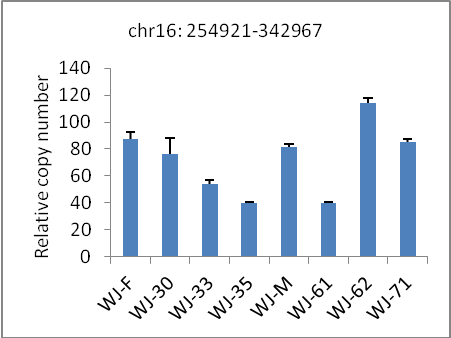

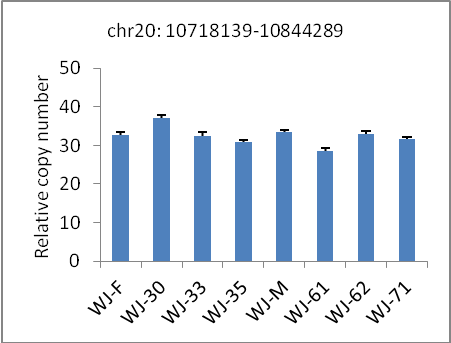


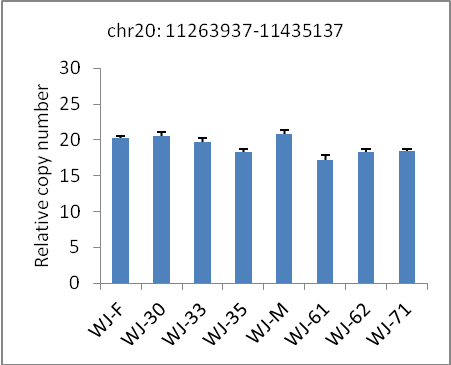

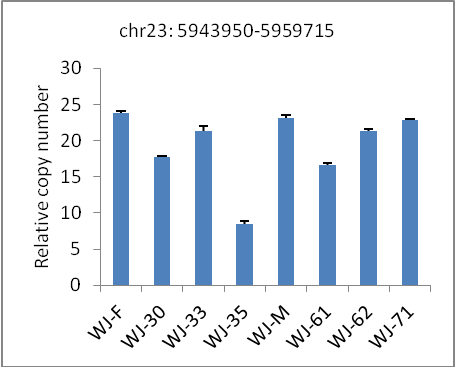


Primers used for qPCR validation.

| CNVR locus | Forward primer (5'→3') | Reverse primer (5'→3') |
| --- | --- | --- |
| chr1: 88675683–88695234 | TTTCCACAGAACTGGCACTGAA | GGATCTCCGTCAGCATATGAATAA |
| chr1: 106296860–106308122 | TGTAATGGAATGAGGTGGCTACA | TCTATGAGGTATGAACAGGAGCAGAT |
| chr1: 145045417–145056781 | CCTTTAGAAAAAAAAGGTCAACATGA | TCAATAATTTAGCTACTGCGACCAA |
| chr1: 167926654–167954794 | TTTATTGTTTAAGGATACATGCAGGAGTA | ACAGGTTGAGTAAATCTGGTCATACG |
| chr2: 12388597–12405194 | TGCCAGTTGGGAGAGATATTTATTT | GATGAAGTCAAGAAGGCTGCAA |
| chr2: 86321304–86335948 | AGCACAACCGAAACAACTACATACA | GGGAGGAGATGCCATTGGA |
| chr2: 97312205–97338589 | CGGCGAATAATCGTGGAAAT | CAAAGGAGGTCCCTGCAGTCT |
| chr2: 106517823–106529907 | AGCAGGACAGCTTGCCAAAT | TGATTGAGCAAGAGATAGAGGAAGAC |
| chr2: 130453299–130471785 | TCGACTCCAATTCTTGAAGATGTG | CTGTGCAGCTGGATGTGGTT |
| chr3: 13793660–13803875 | TCCTTTTCCAGCTCGTACACTGT | TCTGAAGAAAGGTTGAGCTTATACTGA |
| chr3: 107796818–107810140 | TCTTTGTGACGGATGACTTATATGC | GGTAACACTTGGTGCCCACAGT |
| chr4: 19183231–19194128 | CTGTAAATTGGACACTTGTGATCTCA | GCTTTGGCATCATAAAACAAAGAG |
| chr4: 49370878–49386441 | TGTTCCCCTTCCAATTCTGAA | TGCAGGGTCACTCCCTTGA |
| chr4: 88929685–89054302 | GGGCCTCCATCGTGTTTCT | AACTTCTGCCCACAGGCTGTA |
| chr5: 3104188–3138583 | CTAAGCAGACAAGAAAAAAGGCATAG | TTGCGGTGTGCTGTGGAA |
| chr6: 11476973–11511183 | CATTATTAGGCTCTCCAACACAGTGA | CAAAGCAGCAACAACAGCAAA |
| chr8: 27562722–27579422 | CGATCACAAAAGCCTATCTGGAT | CGGCCAAAGGCAATGG |
| chr9: 19750362–19757329 | AGGACCGAAAGTAAATTTCCTACAGA | AATGTAAGGCACTGGGATGAATG |
| chr11: 2674301–2687093 | TCGTTTCAGAGAACCTCCACAGT | AGGACTTGTGGCATCTGAACAA |
| chr13: 15750417–15766190 | GCATCAAATTCTTTGGGAATCAA | TTGCTTAGCCTAAAATAGCCACTTG |
| chr16: 254921–342967 | TGAGGAATGCAGCGTTCATC | TGCGCAGGCTCCCTTTC |
| chr20: 10718139–10844289 | AACAGTGTGTGCCCTTACCTATGTT | GCAGATGATTGAAGAGAGATTTTAAATG |
| chr20: 11263937–11435137 | AGGCATAAGCACAAGAAAAACCA | CAAAGCCTTTGTTAGTCCAACCA |
| chr23: 5943950–5959715 | GCTCTAAATCTAAGCAAGAAACAGACAT | AACAACACCCTGAAAGCCTAAGA |

**Summary of the statistical analysis results for qPCR validation in 24 loci (totally 28 sets).**

| CNVR locus | F-test | Paired T-test | *p* Value | aCGH results for selected sample | | Concordance between qPCR and aCGH results |
| --- | --- | --- | --- | --- | --- | --- |
| chr1: 88675683–88695234 |  | √ | <0.01 | QY-M gain | QY-F normal | Yes |
| chr1: 106296860–106308122 | √ |  | <0.05 | WJ-M normal | WJ-F loss | Yes |
| chr1: 145045417–145056781 | √ |  | <0.01 | WJ-M loss | WJ-F loss | Yes |
| chr1: 167926654–167954794 for QY | √ |  | <0.05 | QY-M gain | QY-F gain | Yes |
| chr1: 167926654–167954794 for WJ | √ |  | <0.01 | WJ-M gain | WJ-F gain | Yes |
| chr2: 12388597–12405194 |  | √ | <0.01 | WJ-M loss | WJ-F loss | Yes |
| chr2: 86321304–86335948 |  | √ | <0.01 | WJ-M gain | WJ-F gain | Yes |
| chr2: 97312205–97338589 | √ |  | <0.01 | QY-M loss | QY-F normal | Yes |
| chr2: 106517823–106529907 | √ |  | <0.05 | QY-M gain | QY-F normal | Yes |
| chr2: 130453299–130471785 |  | √ | >0.05 | QY-M normal | QY-F gain | Yes |
| chr3: 13793660–13803875 | √ |  | <0.01 | WJ-M loss | WJ-F loss | Yes |
| chr3: 107796818–107810140 for QY |  | √ | <0.01 | QY-M loss | QY-F loss | Yes |
| chr3: 107796818–107810140 for WJ |  | √ | <0.01 | WJ-M loss | WJ-F loss | Yes |
| chr4: 19183231–19194128 | √ |  | <0.01 | QY-M gain | QY-F gain | Yes |
| chr4: 49370878–49386441 | √ |  | <0.01 | WJ-M loss | WJ-F loss | Yes |
| chr4: 88929685–89054302 | √ |  | <0.01 | QY-M loss | QY-F gain | Yes |
| chr5: 3104188–3138583 |  | √ | <0.01 | WJ-M loss | WJ-F loss | Yes |
| chr6: 11476973–11511183 |  | √ | <0.01 | WJ-M loss | WJ-F loss | Yes |
| chr8: 27562722–27579422 |  | √ | <0.05 | QY-M loss | QY-F normal | No (QY-M did not show the same variation in this locus) |
| chr9: 19750362–19757329 | √ |  | <0.01 | QY-M loss | QY-F normal | No (QY-F did not show the same variation in this locus) |
| chr11: 2674301–2687093 | √ |  | <0.01 | WJ-M gain | WJ-F normal | Yes |
| chr13: 15750417–15766190 for QY |  | √ | <0.01 | QY-M gain | QY-F gain | Yes |
| chr13: 15750417–15766190 for WJ | √ |  | <0.01 | WJ-M gain | WJ-F gain | Yes |
| chr16: 254921–342967 | √ |  | <0.01 | WJ-M gain | WJ-F gain | Yes |
| chr20: 10718139–10844289 |  | √ | <0.01 | WJ-M gain | WJ-F gain | Yes |
| chr20: 11263937–11435137 | √ |  | <0.01 | WJ-M gain | WJ-F gain | Yes |
| chr23: 5943950–5959715 for QY | √ |  | <0.05 | QY-M gain | QY-F normal | Yes |
| chr23: 5943950–5959715 for WJ | √ |  | <0.01 | WJ-M gain | WJ-F gain | Yes |
